# Pharmaceutical needs at temporary dispensing sites in Suzu City during the subacute phase after the 2024 Noto Peninsula Earthquake: a cross-sectional study of disaster prescription trends

**DOI:** 10.1186/s40780-026-00563-5

**Published:** 2026-03-18

**Authors:** Yukari Koike, Shuji Yamashita, Natsuki Umiji, Aina Kano, Aoi Koketsu, Yuka Nakasato, Momoka Yamashita, Nobuhiro Namaki, Masako Hashimoto, Akihiro Watanabe, Taihei Yamada, Hideki Hayashi

**Affiliations:** 1 Laboratory of Community Pharmaceutical Practice and Science, Gifu Pharmaceutical University, 1-25-4 Daigaku-Nishi, Gifu, 501-1196 Japan; 2 Laboratory of Community Pharmacy, Gifu Pharmaceutical University, 1-25- 4 Daigaku-Nishi, Gifu, 501-1196 Japan; 3 Gifu Pharmaceutical Association, 4-5 Kokonoe-cho, Gifu, 500-8146 Japan; 4 Ishikawa Pharmaceutical Association, 25-10 Hiromachi-i, Kanazawa, Ishikawa 920-0032 Japan; 5 Department of Crisis Management Medicine, Hyogo Medical University, 1- 1 Mukogawa-cho, Nishinomiya, Hyogo 663-8501 Japan; 6 Department of Emergency, Disaster and Critical Care Medicine, Hyogo Medical University, 1-1, Mukogawa-cho, Nishinomiya, Hyogo 663-8501 Japan

**Keywords:** Mobile pharmacy vehicle, 2024 Noto Peninsula Earthquake, Disaster medicine, Pharmaceutical demand, Essential medicines lists

## Abstract

**Background:**

This study examined pharmaceutical needs in the subacute phase following the 2024 Noto Peninsula Earthquake, using dispensed prescriptions as a proxy. We described demand patterns, the dispensing timeline amid early shortages, and the alignment between observed requirements and four disaster medicine lists.

**Methods:**

We conducted a retrospective cross-sectional analysis of prescriptions dispensed in disaster-affected areas of Japan (primarily Suzu City) between January 7 and 13, 2024. A total of 236 prescriptions were included and categorized into major therapeutic classes. We separated regular prescriptions from as-needed (pro re nata, PRN) prescriptions. The dispensing interval was defined as the number of days from prescription issuance to dispensing. Coverage was calculated by comparing dispensed medicines against four disaster medicine lists, including the Japanese Medical Association Team (JMAT) Carry-on Medicine List.

**Results:**

Cardiovascular, central nervous system, and gastrointestinal drugs were the most commonly prescribed medicines. Early shortages delayed dispensing until wholesale supplies resumed on January 11. Regular and PRN prescriptions accounted for 41.9% and 53.8% of all prescriptions, respectively. However, PRN prescriptions were dispensed in fewer days. Coverage of the four disaster medicine lists ranged from 41.8% to 65.9%, with the highest coverage for the JMAT Carry-on Medicine List.

**Conclusions:**

This study clarified the characteristics of pharmaceutical needs in Suzu City during the subacute phase, albeit within a limited scope based on the activities of specific medical support teams. Observed pharmaceutical requirements only partially overlapped with existing lists. Portable medicine lists, regional stockpiles, and local formularies tailored to community needs could improve responsiveness. Interim measures, such as temporary services and mobile support, helped bridge the gap in medicine access before supply chain recovery. In this setting, mobile pharmacy vehicles potentially played a central role until normal distribution resumed. These data can inform future disaster medicine planning.

## Background

The Noto Peninsula Earthquake, which occurred on January 1, 2024, caused major damage to the area (Table 1). Suzu City has a total population of approximately 13,000, with an aging rate (65 years and older) of 51.7%. Furthermore, single-person or couple-only elderly households account for 44.2% of the total households, reflecting a significantly aged demographic (2020 Census) [1]. As a result, many clinics in the northern part of the Noto Peninsula, near the epicenter, were closed. Furthermore, no health insurance pharmacies existed in Suzu City, making it necessary to provide medications to affected residents urgently. In response, the Gifu Pharmaceutical Association, after receiving a request for assistance from the Ishikawa Pharmaceutical Association through the Japan Pharmaceutical Association, dispatched a Mobile Pharmacy Vehicle (MPV) to the Suzu City Health and Welfare Center (referred to as “the Center”). The MPV is a mobile pharmacy unit developed in Japan specifically for disaster response. It is designed to be rapidly deployed to areas where medical institutions and pharmacies have been damaged, providing an essential supply of pharmaceuticals and pharmaceutical care to the affected population. The support team arrived on January 7 and accompanied the mobile medical team in visiting evacuation centers to provide prescription support for doctors, dispense medications, and supply medication instructions. After the temporary dispensing station was established within the Center, the support team mainly provided prescription dispensing services for disaster-related prescriptions issued by the mobile medical team until the Hiroshima MPV took over operations on January 14.

**Table 1:** Summary of damage caused by the 2024 Noto Peninsula Earthquake

Occurrence date/time	January 1, 2024, 16:06
Maximum magnitude	7.6
Maximum intensity	7
Damage situation in Ishikawa Prefecture	
Deaths	623
Severe injuries	395
Minor injuries	876
Houses completely destroyed	6,163
Houses partially destroyed	18,713
Evacuees (January 2, first evacuation)	40,688
Evacuation centers (maximum)	423

Damage from the 2024 Noto Peninsula Earthquake (Cabinet Office) (As of August 5, 2025, 16:00)

Status of Shelter Operations for the 2024 Noto Peninsula Earthquake (Cabinet Office) (April 15, 2024)

Several research reports on disaster relief activities have been published, not only for the 2024 Noto Peninsula Earthquake, wherein the authors also provided relief, but also for the Great East Japan Earthquake, the Niigata Chuetsu Earthquake, the 2016 Kumamoto Earthquake, and others [2–6]. These reports have been utilized in actual disaster responses through subsequent disaster drills and manual creation. Of these reports, research on disaster prescriptions issued in disaster-stricken areas has been published for the Great East Japan Earthquake and the 2016 Kumamoto Earthquake [7–10]. However, to the best of our knowledge, no research has been conducted on the 2024 Noto Peninsula Earthquake. Furthermore, whereas medications for disaster relief are selected from lists published by multiple academic societies and organizations, including the Japanese Association for Disaster Medicine and Japanese Medical Association Team (JMAT) [11–14], there have been no reports evaluating their usefulness compared to medications prescribed in actual disasters since the publication of each list. Therefore, in this study, we investigated the actual demand for medicines dispensed by the MPV operating at the center or the temporary dispensing office within the center in the early stages following the 2024 Noto Peninsula Earthquake. We also aimed to evaluate the usefulness of this data by comparing it to the lists of medicines to be stocked in emergencies published by various organizations.

## Methods

### Survey subjects and data collection period

Data on the inventory management of pharmaceuticals dispensed from the MPV or the temporary dispensing site established within the Suzu City Health and Welfare Center were used, based on disaster prescriptions issued between January 6 and January 13, 2024, following the 2024 Noto Peninsula Earthquake.

### Survey items

Data on prescription acceptance date, dispensing completion date, drug name (as written by the physician), drug name (dispensed), number of dispensed, prescription changes, and deletions were extracted from the prescription dispatch data. The number of disaster prescriptions dispensed and the number of days were tallied, and the dispensed pharmaceuticals were categorized by efficacy. The Japan Standard Commodity Classification Code (JSCC), established by the Ministry of Internal Affairs and Communications, was used to categorize the medicines.

### Classification by prescription purpose

Using pharmaceutical inventory management data for disaster prescriptions filled during the target period, we classified each prescription as either a regular prescription or an as-needed (pro re nata, PRN) prescription. PRN prescriptions were defined as oral medications with a prescription period of < 14 days and containing three or fewer medications, and topical medications. We also compared the number of days required for regular prescriptions and PRN prescriptions.

### Comparisons with pharmaceutical lists

We compared the pharmaceutical inventory data for drugs used immediately after the 2024 Noto Peninsula Earthquake with pharmaceuticals on lists issued by domestic and international organizations, which are expected to serve as reference materials when selecting items to carry in the event of a disaster. The list comprises four documents: the List of Essential Medicines for Hyper-Acute Phase in Disasters (excluding medicines for emergency medical treatment by DMATs) Revised Edition 2024 (Japanese Association for Disaster Medicine), the Revised Disaster Preparedness Manual for Pharmacists (Research Team, Ministry of Health, Labour and Welfare (MHLW) Scientific Research FY2023 “Research on Disaster Response by Pharmacists and Pharmacies”), the JMAT Carry-on Medicine List ver. 3.0 (Japan Medical Association), and The selection and use of essential medicines, 2025: World Health Organization (WHO) Model List of Essential Medicines, 24th list.

Comparison with each medicine list was assessed using strict match and extended match methods (allowing therapeutic substitutions). The WHO Essential Medicines List was grouped into five categories: (1) Strict match A (the same drug as the dispensed medicine is listed); (2) Strict match B (can be addressed by changing the specifications; a drug with the same ingredients is listed); (3) Expanded match A (the smallest category of each category has the same primary mechanism but different ingredients); (4) Expanded match B (the smallest category of each category has a different primary mechanism); (5) No match (no matching drug on the list; categories other than 1–4). Additionally, drugs classified as 1–3 were defined as those listed in the relevant categories. The coverage rate for each list was calculated using the total number of drugs per item as the denominator.

### Statistical analysis

The chi-square test was used to compare differences between two groups. The statistical software used was IBM SPSS version 29 (IBM Corp., Armonk, NY, USA), and the significance level was set to *p* < 0.05.

### Ethical considerations

This study was approved by the Gifu Pharmaceutical University Research Ethics Committee through a central review in accordance with the ethical guidelines for medical research involving human subjects (Approval Number: 7–21).

## Results

### Number of prescriptions issued and days required to complete dispensation

**Fig. 1:**
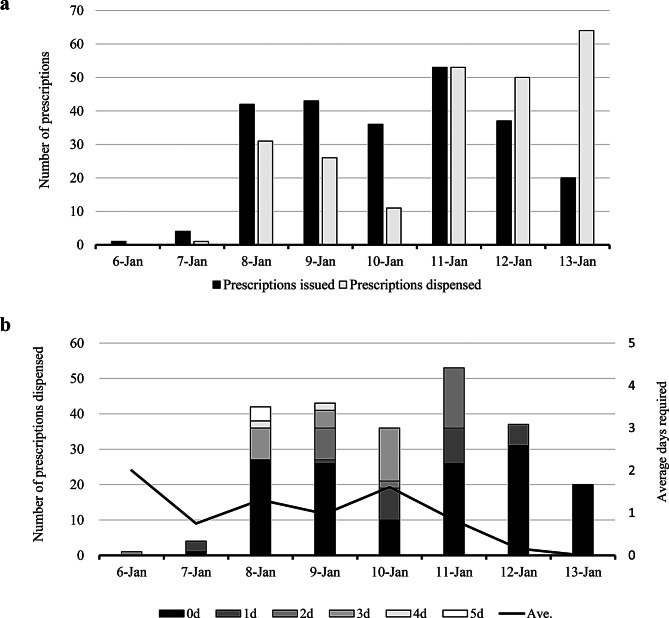
Number of prescriptions issued and days required for dispensation. **a**: Trends in the number of disaster prescriptions issued and dispensed. **b**: Trends in the number of days required for dispensation

Figure 1a shows the number of prescriptions issued and dispensed by day during the study period. Altogether, 236 prescriptions were issued, of which 73 required some substitutions, such as a brand name versus a generic, dosage form, or standard. Initially, the number of prescriptions dispensed fell below the number of prescriptions issued. However, after pharmaceutical supplies from wholesalers resumed on January 11, the number of prescriptions dispensed rapidly increased. Figure 1b shows the number of days required for prescriptions issued during the disaster period and the average number of days required for dispensation. The maximum number of days required was five, the average number of days required during the period was 0.89, and the average number of days required for each day was less than two days. Although the number of prescriptions issued on January 11 was the highest during the study period, no prescriptions required more than three days for dispensation. Furthermore, by January 13, all prescriptions were dispensed on the same day they were prescribed.

**Fig. 2:**
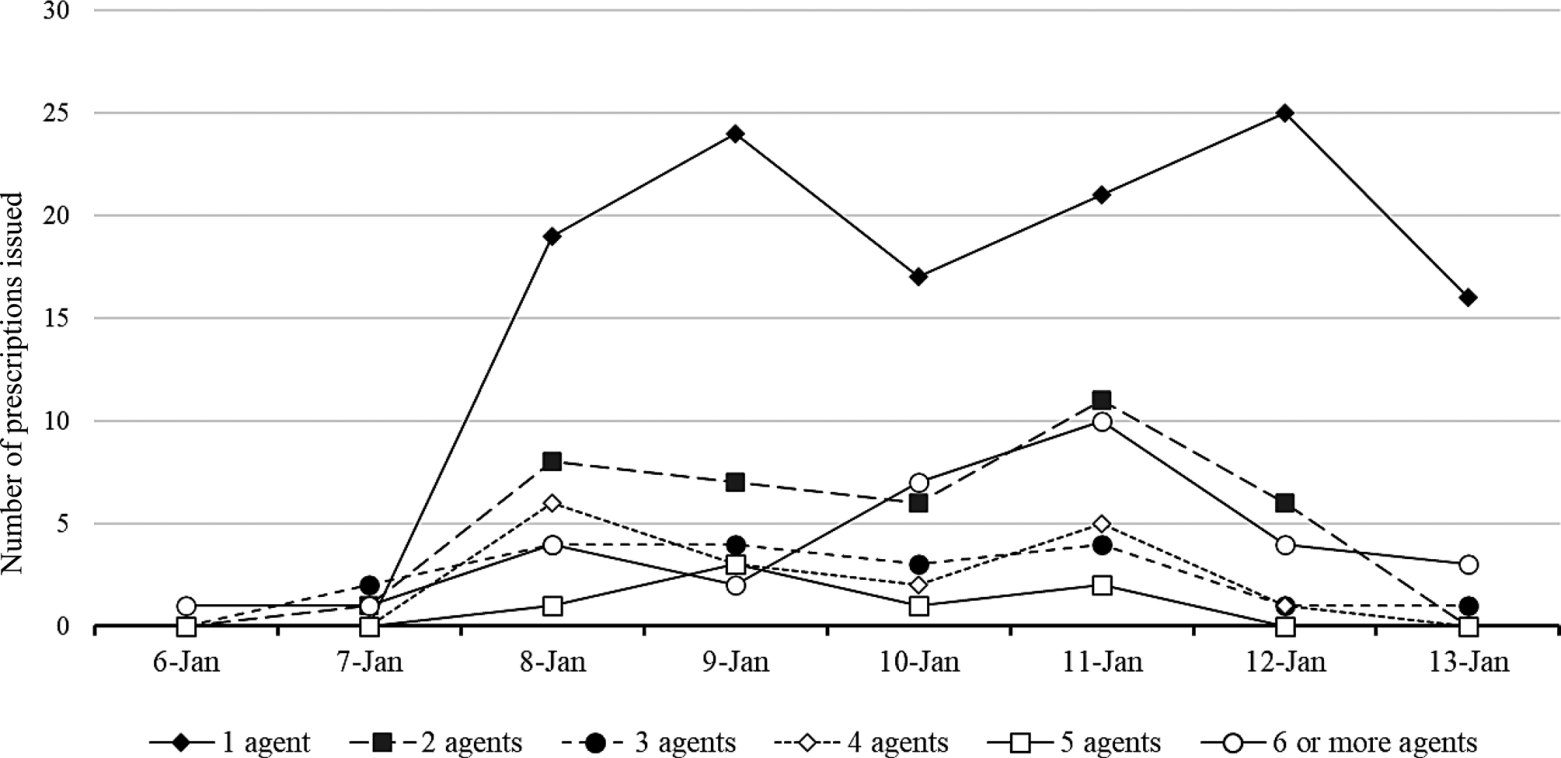
Trends in the number of drugs dispensed per prescription

The number of medications prescribed per prescription is also shown by day (Fig. 2). Prescriptions containing six or more medications were classified into a single category, commonly known as polypharmacy. Most prescriptions contained a single medication. The proportion of prescriptions containing six or more medications tended to be higher in the latter half of the study period.

### Therapeutic classification of prescription drugs

**Fig. 3:**
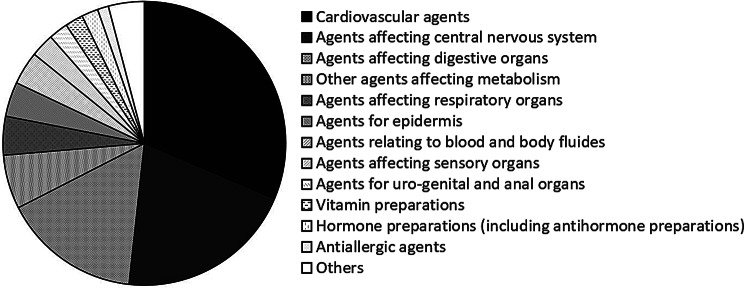
Demand status by therapeutic category

**Fig. 4:**
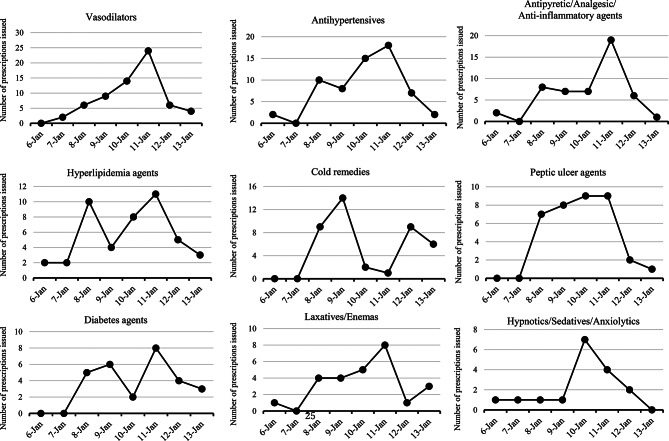
Trend in demand status by therapeutic category

Figure 3 shows the categorization of prescription drugs by therapeutic group based on the subclassification of the Standard Product Classification for Japan. If the total number was < 5, these were combined into a single category, “Other.” Twenty-one drug classes were prescribed, with the most common being cardiovascular, central nervous system, and gastrointestinal medications. Medications were further subdivided into subtypes; central nervous system medications included anxiolytics/hypnotics, antipyretics/analgesics, and general cold medications. Figure 4 shows time-dependent changes in prescription numbers for the nine most commonly prescribed classes.

### Classification of regular and PRN prescriptions

**Fig. 5:**
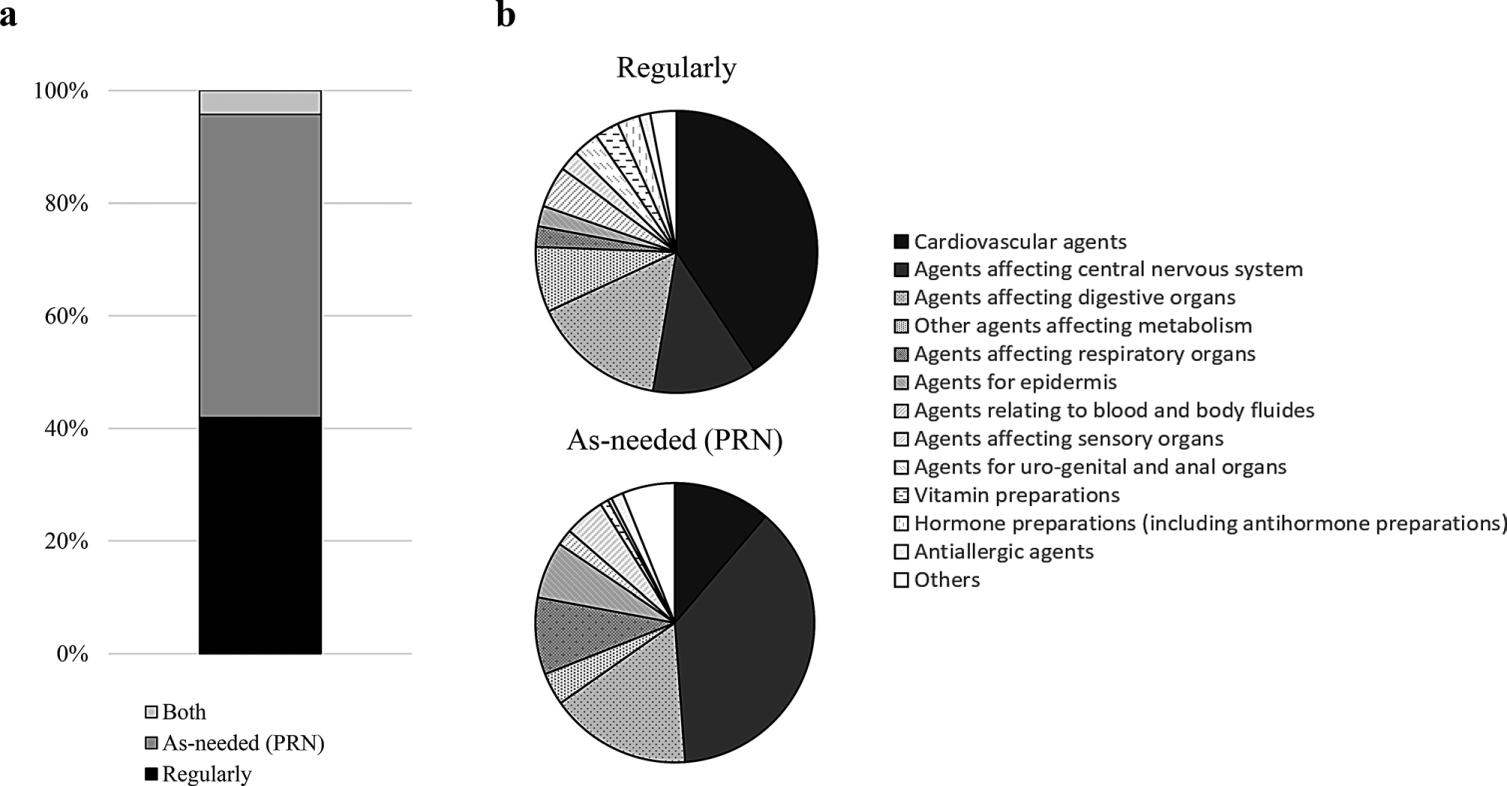
Characteristics of the ratio of regular to temporary prescriptions during the activity period. **a**: Overall ratio. **b**: Breakdown by therapeutic category. Prescriptions classified as “Both” were added to each category (b)

Disaster prescriptions issued during the study period and filled at MPVs or temporary pharmacies were categorized by issue date as regular, extraordinary, or both. The overall percentage was also documented (Fig. 5a). For regular prescriptions, cardiovascular agents, digestive organ agents, and central nervous system agents were the most common. In contrast, for PRN prescriptions, central nervous system agents, digestive organ agents, and cardiovascular agents followed in descending order of frequency (Fig. 5b).

**Fig. 6:**
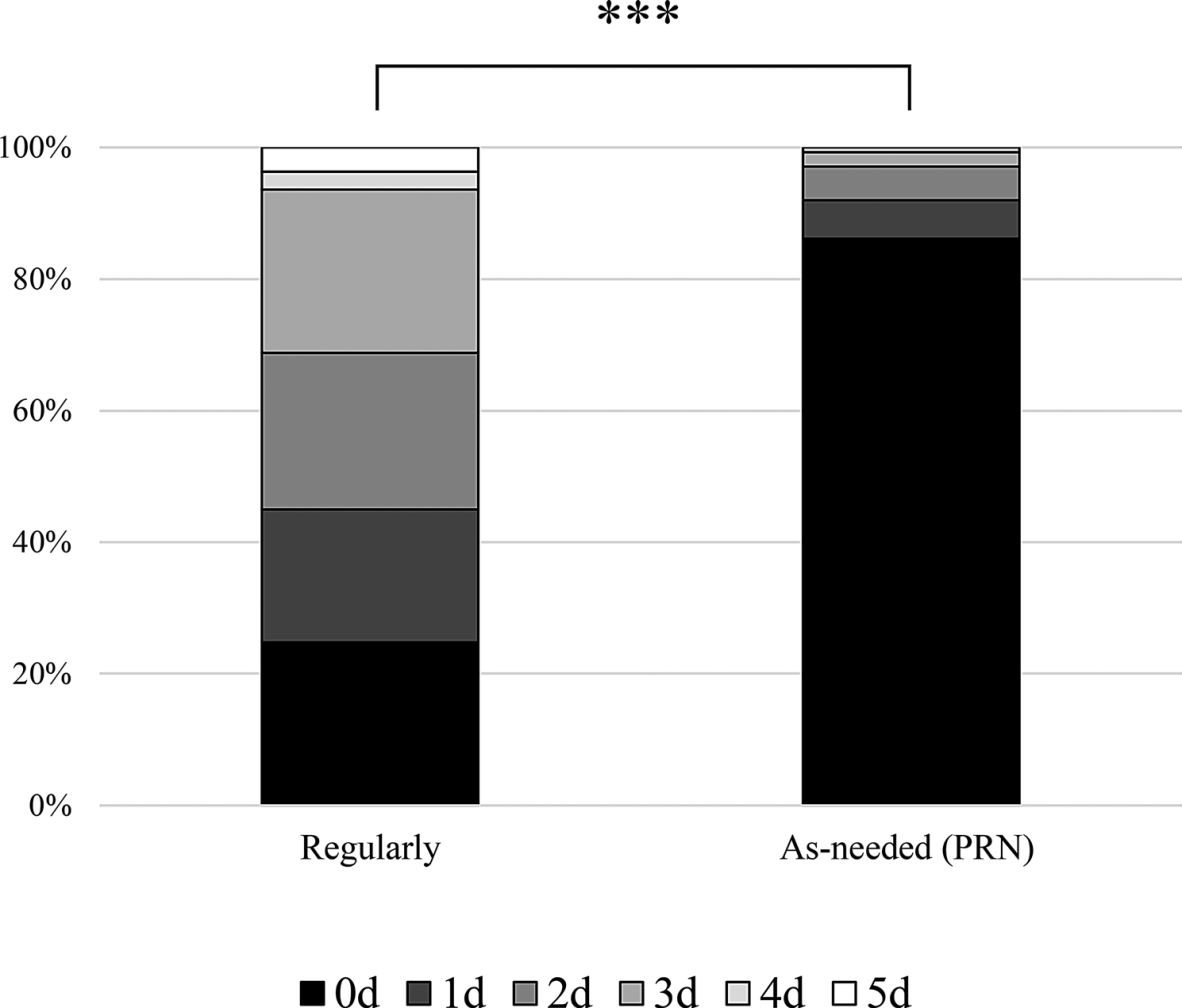
Comparison of regular vs. as-needed (PRN) prescription ratios. Prescriptions classified as “Both” were added to each category. 0 days vs. Others, χ² test, ***P < 0.001. PRN, pro re nata

Both regular and PRN prescriptions were issued each day since January 8. Regular prescriptions accounted for 41.9% of the total, and PRN prescriptions comprised 53.8%. Figure 6 shows the regular and PRN prescriptions by the number of days required. Comparing the proportion of prescriptions requiring 0 days (completed on the same day) and other (one or more days) between the two groups, the proportion of prescriptions requiring 0 days was significantly higher for ad hoc prescriptions (*p* < 0.001).

### Classification of responses to prescription drug substitutions based on drug lists

**Fig. 7:**
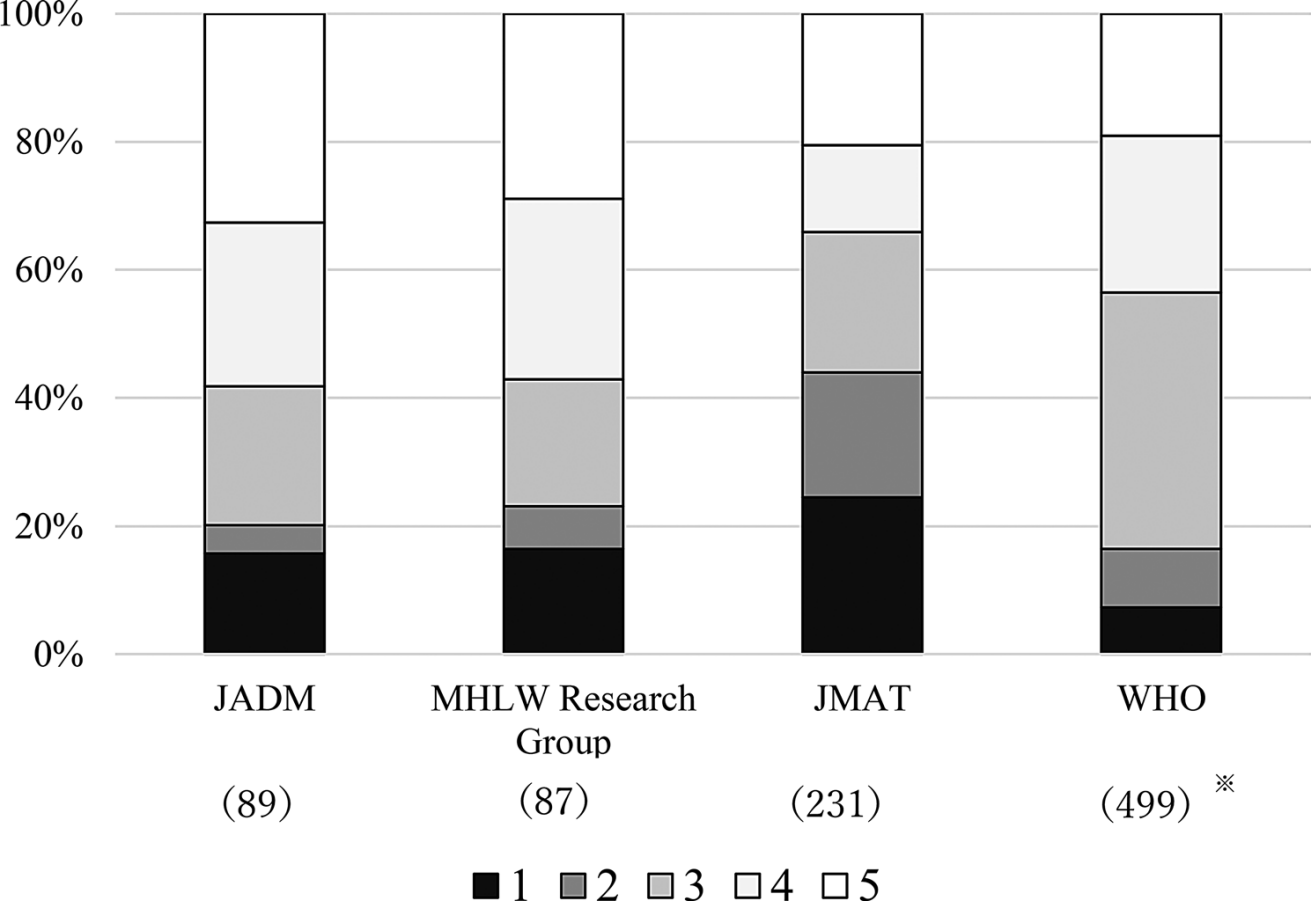
Corresponding categories for prescribed drugs in each list. Numbers in parentheses indicate the number of items listed. ※Excludes items listed as alternative candidates. (1) Strict match A, (2) Strict match B, (3) Extended match A, (4) Extended match B, (5) No match (categories other than 1–4). JADM: List of Essential Medicines for Hyper-Acute Phase in Disasters (excluding medicines for emergency medical treatment by DMATs) Revised Edition 2024. MHLW Research Group: Revised Disaster Preparedness Manual for Pharmacists (Research Team, Ministry of Health, Labour and Welfare Scientific Research FY2023 “Research on Disaster Response by Pharmacists and Pharmacies”). JMAT: JMAT Carry-on Medicine List ver. 3.0 (Japan Medical Association). WHO: The selection and use of essential medicines, 2025: WHO Model List of Essential Medicines, 24th list

Figure 7 categorizes the prescriptions prescribed during the study period into five categories based on the assumed drug supply for each drug list. The coverage rates for the four lists based on this definition were 41.8% for the Japanese Society of Disaster Medicine, 42.9% for the MHLW Scientific Research Group, 65.9% for the JMAT Carry-on Medicine List, and 56.4% for the WHO Essential Drug List. The coverage rate was highest for drug supplies based on the JMAT Carry-on Medicine List. The lists from the Japanese Society of Disaster Medicine and the MHLW Scientific Research Group displayed similar trends. Furthermore, excluding items listed as potential substitutes, the WHO Essential Drug Model had the most drug items listed, whereas the MHLW Scientific Research Group’s list had the fewest.

## Discussion

In this study, we investigated the pharmaceutical demand in the subacute phase following the Noto Peninsula Earthquake in disaster prescription delivery data from the MPV and temporary dispensing offices at the Suzu City Health and Welfare Center. Based on existing literature defining the subacute phase as the period from one week to approximately one month post-disaster [2, 12], we identified the study period as the subacute phase.

In total, 236 prescriptions were filled during the study period. When MPV activities began, the number of prescriptions dispensed was lower than the number issued. After January 10, the average number of days required from prescription acceptance to dispensing completion gradually decreased. No prescriptions required more than four days to be dispensed. Pharmaceutical supplies from wholesalers to the Health and Welfare Center were resumed on January 11. By January 13, all prescriptions were dispensed on the same day they were prescribed. This finding likely reflects improved distribution and a more stable workflow, resulting in increased supply speed. A disaster support agreement had been established between Ishikawa Prefecture and the Ishikawa Pharmaceutical Wholesalers Association since November 1996 [15]. According to Ministry of Health, Labour and Welfare records, wholesale pharmaceutical supplies were generally available for next-day delivery from at least January 5 onwards [16], and were indeed being supplied to hospital within Suzu City. However, despite official notifications issued to facilitate the redistribution of pharmaceuticals among medical institutions and local governments [17], it took 10 days from the onset of the disaster to begin supplying medications to the MPV and the temporary dispensing site at the Center. This delay, particularly given the absence of community pharmacies in the city, suggests that in addition to geographical factors such as the city’s location at the tip of the peninsula and road damages, factors related to the Command and Control system for establishing a pharmaceutical supply infrastructure were significant contributors.

The medications stocked in the MPV were coordinated and supplied by the Ishikawa Pharmaceutical Association. As of January 10, immediately before the resumption of wholesale pharmaceutical supply, the inventory consisted of 56 oral and 24 topical medications. Leveraging its mobility, the MPV not only responded to disaster prescriptions brought directly to the unit but also accompanied medical teams on mobile clinics at nine locations over a three-day period. Between the start of operations and January 10, when the temporary dispensing site was established at the Center, the MPV dispensed prescriptions issued by at least 13 different medical teams (out of a total of at least 26 teams during the entire study period). Since no other facilities or teams in Suzu City were capable of dispensing disaster prescriptions during this period—apart from the MPV and the Center’s temporary site—it is highly likely that the MPV handled the majority of the disaster prescriptions issued by medical teams active in the city. Thus, the MPV potentially played a central role in the pharmaceutical supply chain until wholesale deliveries resumed and the temporary dispensing site was fully operational.

Whereas the most common number of medications per prescription was one, a trend toward multi-drug prescriptions with six or more medications emerged in the latter half of the period. This finding implied that as time passed after the disaster, patients with chronic diseases increasingly obtained disaster prescriptions due to the depletion of their regular medication stocks. Previous disasters have also demonstrated an increase in prescriptions for conditions requiring long-term medication, such as hypertension, diabetes, and dyslipidemia, as conditions transition to the chronic phase [12].

By drug category, cardiovascular, central nervous system, and digestive medications dominated the top categories. Previous reports have shown similar trends in Minamiaso Village and during the latter half of the Great East Japan Earthquake [7, 8]. In the present study, cardiovascular agents were the most prescribed for regular prescriptions, whereas central nervous system agents were the most frequent for PRN prescriptions, indicating a clear difference in medical needs between the two. This trend for central nervous system agents is likely attributable to the high demand for multi-symptom cold remedies and antipyretic analgesics.

Furthermore, this study showed that 41.9% of prescriptions were regular and 53.8% were PRN prescriptions. The proportion completed on the same day was significantly higher for PRN prescriptions, suggesting that the number of days required increased with the proportion of regular prescriptions. Over approximately one week, 236 prescriptions were filled, comprising 273 different medications. Thus, theoretically, each patient was prescribed at least one different medication. Regular prescriptions typically comprise a larger variety of medications than special prescriptions. One contributing factor may be that prescriptions presumably for regular medications accounted for over 40% of the total, since regular prescriptions typically involve a greater variety of drugs than PRN prescriptions. Whereas an increase in the number of drugs provides patients with peace of mind by ensuring they can obtain their “usual” medications, it also raises the risk of increased waste after the end of support and a higher workload, such as for purchasing new medications and providing alternative dispensing services, resulting in increased time required for dispensation.

To mitigate these risks, measures such as creating a reference list for selecting portable medications, establishing regional stockpile plans, and developing regional formularies, and dispensing medications outside the disaster-affected area should be considered. Until local medical institutions and wholesalers resume providing supplies, support teams, under the direction of the local Health, Medical, and Welfare Command Center, must address both acute PRN prescriptions and routine regular prescriptions. Therefore, support teams may need to select stocked medicines based on the disease structure, environmental conditions, and seasonal variations in the affected area. Additionally, local stockpiling plans are necessary. Furthermore, utilizing regional formularies [18] and having patients maintain a 7–10 day supply of their regular medications are suggested countermeasures. Regional formularies are regional collections of drugs and their use guidelines, including those deemed optimal from a comprehensive perspective, encompassing efficacy, safety, and cost-effectiveness, through collaboration among local physicians, pharmacists, and other healthcare professionals and their related organizations [18]. However, there are several concerns. Whereas formularies are increasingly developed at the medical institution level, according to a survey by the MHLW [19], only 12 prefectures have created at least one regional formulary, and the development scope remains limited. Collaboration between medical associations, pharmacist associations, and other related organizations is essential for developing regional formularies. Building the necessary relationships for formulary development could also have a positive impact on collaboration during disasters, so this will hopefully be expanded to more regions. Prescribing patients with a reserve supply of their regular medication during outpatient visits requires an assessment of the patient’s self-management capabilities. Additionally, some medications, such as psychotropic drugs requiring management [20] and high-risk medications requiring intermittent administration, may be inappropriate for issuance as backups. According to the Medical Care Regulations [21], “dosages must be based on the foreseeable duration of need,” leading some to interpret this as a limitation to prescribing backups [22]. Furthermore, the implementation of telemedicine, online medication counseling, and pharmaceutical supply systems utilizing drones could potentially mitigate these risks. However, because disaster victims requiring regular medications are generally elderly, there are significant psychological and environmental hurdles to adopting online medical and pharmaceutical services. Additionally, the supply of medications via drones faces challenges such as the administrative burden of legal procedures and the lack of concrete operational frameworks. Therefore, further studies are necessary to address these issues for future practical application.

Comparing medications in demand during the study period with the medication lists issued by each organization, the JMAT Carry-on Medicine List had the highest coverage rate at 65.9%, followed by the WHO Essential Medicines Model, the MHLW Scientific Research Group, and the Japanese Society of Disaster Medicine. JMAT primarily focuses on activities in evacuation centers and other settings during the post-acute phase, which aligns well with the disaster phase examined in this study. However, since the medications available at the MPV and the temporary dispensing site at the Center were inherently limited, the prescription patterns observed during the study period were likely influenced by the stock availability. The Disaster Medicine List provides a list of medications considered minimally necessary immediately after a disaster (up to around 10 days after the disaster). The MHLW Scientific Research Group’s list was compiled based on the Disaster Medicine List, so the results should be interpreted with caution. Furthermore, although the WHO list had the most items, it did not have the highest coverage rate. This finding might be attributed to the selection criteria that were not limited to medicines that are required during disasters, and the inclusion of medicines not typically used in Japan.

This study had several limitations. It is important to note that these findings are highly specific to this region, given the unique circumstances such as Suzu City’s geographic location at the tip of the peninsula and the absence of community pharmacies within the city. Due to the existence of medical teams distributing on-hand medications, outpatient services (limited to emergencies) at the disaster base hospital in the city, as well as the short eight-day period covered, it was not possible to assess the pharmaceutical demand for disaster prescriptions in Suzu City following the Noto Peninsula earthquake accurately. Regarding the number of disaster prescriptions issued, a potential limitation is that because the disaster occurred during the year-end and New Year holiday period, some patients may have received longer-term prescriptions for their regular medications than usual, possibly reducing the apparent demand during the study period. Our classification of prescription change responses was based on definitions for each response and drug category, so opinions on actual responses might vary. Direct comparisons between the WHO Essential Medicines Model and other lists are challenging due to differences in strict classification definitions, and care must be taken when interpreting the results.

## Conclusions

In this study, by comparing pharmaceutical demand with various disaster medicine lists, we have clarified—within a limited scope based on the prescription trends of medical support teams—the characteristics and challenges of medication needs in Suzu City during the subacute phase of the 2024 Noto Peninsula Earthquake. This study will be a valuable reference for developing medicine and stockpile lists for future disaster responses.

## Data Availability

The datasets used and/or analyzed during the current study are available from the corresponding author on reasonable request.

## Abbreviations

JMAT: Japanese Medical Association Team
JSCC: Japan Standard Commodity Classification Code
MHLW: Ministry of Health, Labour and Welfare
MPV: Mobile Pharmacy Vehicle
PRN: pro re nata
WHO: World Health Organization
